# Development of the Family Togetherness Scale: A Mixed-Methods Validation Study in Kenya

**DOI:** 10.3389/fpsyg.2021.662991

**Published:** 2021-06-08

**Authors:** Eve S. Puffer, Ali Giusto, Amber D. Rieder, Elsa Friis-Healy, David Ayuku, Eric P. Green

**Affiliations:** ^1^Department of Psychology and Neuroscience, Trinity College of Arts and Sciences, Duke University, Durham, NC, United States; ^2^Duke Global Health Institute, Duke University, Durham, NC, United States; ^3^Department of Behavioral Sciences, College of Health Sciences, School of Medicine, Moi University, Eldoret, Kenya

**Keywords:** family functioning, assessment, diagnostic accuracy, Kenya, low- and middle income countries, measure validation, global mental health, child mental health

## Abstract

Family functioning is an important target of clinical intervention and research given its close ties with mental health outcomes of both children and adults. However, we lack family functioning measures validated for use in many low- and middle-income country (LMIC) settings. In this mixed-methods prospective diagnostic accuracy study, we first used formative qualitative data to develop an extensive battery of screening items to measure family functioning in Kenya. We then recruited 30 Kenyan families (*N* = 44 adults; 30 youth aged 8–17 years) to complete the questionnaires and participate in clinical interviews conducted by local interviewers. Quantitative and qualitative analyses were then conducted to select a subset of screening items that balanced conceptual understanding of family distress with diagnostic efficiency and accuracy to yield a brief but valid scale. The final index test consisting of 30 items correctly identified distressed families in 89% of cases according to adult-report and 76% of cases according to child-report. The optimal cutoffs are associated with estimates of sensitivity/specificity of 0.88/0.90 and 0.75/0.77 for adult-report and child-report measures, respectively. The final measure—the *Family Togetherness Scale (FTS)*—assesses global family functioning, including items related to family organization, emotional closeness, and communication/problem-solving. In addition to general items, the scale also includes items explicitly assessing family responses to stressors common in LMIC settings. Results establish a strong rationale for larger-scale validation studies.

## Introduction

A major goal in global mental health is the identification of assessment tools that accurately capture the emotions, behaviors, and functioning of people across contexts and cultures (Gureje et al., 2020). This is complex, as the social and structural environment surrounding an individual influences how they perceive and communicate about psychosocial experiences, ranging from mental health symptoms to interpersonal relationships (Kohrt et al., 2011). This calls for attention to validity and reliability of measures across populations and for openness to developing new assessment tools for specific subpopulations.

Strides have been made to develop rigorous methods for the development and adaptation of mental health measures across settings, including low- and middle-income countries (LMICs; Van Ommeren et al., 1999; Bolton, 2001; Weaver and Kaiser, 2015). The majority of this work, however, has focused on assessing individual-level mental health. Less has been done related to assessment of interpersonal relationships, including family relationships, despite the growing number of family-level interventions. We aim to address this gap by developing and evaluating a measure of family functioning in Kenya designed to be adapted and used in LMIC settings.

### Importance of Measuring Family Functioning

Family functioning encompasses the social and structural characteristics of the family environment, referring to a wide range of constructs, such as cohesion and conflict, adaptability, problem solving, communication, roles, and affective responsiveness (Olson, 2014). Evaluating family functioning is important for contextualizing family influences on psychopathology, informing culturally sensitive treatment, engaging family support, and evaluating family interventions. Family functioning also influences multiple outcomes of clinical interest, including individual mental health. Studies from both high-income countries (HICs) and LMICs consistently show associations between child mental health symptoms and family conflict, harsh parenting, and unsupportive interactions (Repetti et al., 2002; Khasakhala et al., 2013). Conversely, positive family functioning can be protective (Knerr et al., 2013). Converging evidence has driven growth in family interventions in LMICs (Knerr et al., 2013; Pedersen et al., 2019), highlighting the need for contextually-relevant measures of family functioning.

### Challenges and Cultural Considerations in Measuring Family Functioning

Family systems are dynamic, continually evolving over time, and the construct of family functioning is multi-faceted, complicated by the ways that dyadic relationships contribute to the overall functioning of the system. For assessment, cultural- and context-specific influences on family functioning also then pose challenges for developing appropriate measures relevant across different contexts. Variation in beliefs and practices can manifest in ways that can strengthen family systems, impede family functioning, or both. Although impossible to identify all factors influencing families across contexts, it is prudent to consider some of the most salient across cultures.

Three important influences in many LMICs include: (a) gender roles, (b) norms related to family structure and centrality of the family to identity, and (c) exposure to poverty and other community-level adversities. Items on assessments should take these into account, ensuring that items capture the impacts that these factors can have on family functioning. Concerning gender, many societies, including in LMICs, have patriarchal norms placing men in power (see, for example, Wamue-Ngare and Njoroge, 2011)—norms that have been associated with risk for intimate partner violence and child maltreatment (Jewkes et al., 2015; Klevens and Ports, 2017). While a traditional lens—men as providers and women as caretakers—may not be inherently negative, how gender norms affect families is complex when expectations are rigid. Measuring clarity and satisfaction related to roles becomes essential. Second, culturally-influenced norms related to family structure and the importance of family for one's identity can affect family interactions and their impact on individuals. Broadly speaking, average family size is larger in many LMICs, with extended families and multiple generations sometimes living in closer proximity and sharing responsibilities (Bornstein et al., 2016; Griffith and Keane, 2018). Underlying these norms is the influence of collectivism in which identity can be strongly influenced by group membership. And having an identity strongly grounded in family can be a source of support or stress (Griffith and Keane, 2018). For assessment, measures should be amenable to multiple reporters and flexible enough to capture challenges arising from complex, multi-generational, dynamics.

Lastly, but perhaps most salient, poverty confers a cascade of consequences. As the Family Stress Model posits, economic hardship can strain family functioning, impeding a family's ability to respond to stressors (Conger and Donnellan, 2007). In LMICs, poverty often permeates all aspects of family life and can undermine resource sharing; increase disempowerment and conflict; and contribute to violence (Mosoetsa, 2011). Positive family environments, however, may buffer some of poverty's consequences (Bhana and Bachoo, 2011). Assessment tools should include items to capture family coping with stressors of poverty.

### Family Functioning Measurement in LMICs

There are a range of measures validated for measuring family functioning in HICs but very few in LMICs (Hinton et al., 2019). Among family measures used most commonly in LMICs, many evaluate specific dyadic relationships (e.g., parent–child), and those that assess the family system are sometimes narrow in scope (e.g., measuring only communication). Fewer focus on a global understanding of family functioning—the construct of interest in this study. When measuring broader family functioning, researchers have typically either transported a measure developed in HICs, with varying levels of adaptation, or developed a new measure for a specific setting. While both can lead to high-quality measures, most tools to date have very limited evidence of validity and reliability, stemming primarily from lack of resources for validation studies and lack of validation methods that are a good fit for family measures.

A review of family interventions in LMICs provides a helpful look at studies measuring family functioning in these contexts (Pedersen et al., 2019). We reviewed each study that measured more global aspects of family functioning. Among those using measures transported from HIC, we found frequent reporting on reliability statistics of internal consistency or inter-rater reliability, as well as information on basic forward–backward translation. Few measured construct equivalence or content validity (for an exception, see Türkdogan et al., 2018). Among studies using locally-derived measures, they often reported developing items based on formative qualitative data, with consideration of culture-specific values, norms, beliefs, language, and idioms of distress (for examples, see Betancourt et al., 2017, Puffer et al., 2016). Most of these also reported internal reliability statistics, but rarely construct validity.

Across measures represented in the review (Pedersen et al., 2019), none had reported evidence of criterion validity—an important limitation, as criterion validity can be especially valuable in providing cut-off scores to determine thresholds indicating clinical significance of problems. Across the field of global mental health, studies that do attempt to assess criterion validity also are often limited by comparing with gold standards validated only in HIC. To address these challenges, researchers have developed creative, community-based methods that do not rely on diagnoses by professionals or previously-validated scales; for instance, Bolton (2001) have used rapid ethnography to identify locally-relevant syndromes and symptoms and triangulated self- and other-report to identify individual mental health problems in LMICs (Bolton, 2001). Parts of this “known groups” methodology is also present in this study. The approach has limitations, however, and does not apply directly to validating family-level measures focused on capturing relationship dynamics in the broader family system.

### The Present Study

In this study, we aim to address two gaps: (1) the lack of validated assessment tools for measuring family functioning that are culturally- and contextually-relevant for Kenya and potentially other LMICs; and (2) the lack of feasible methodology options for validating family measures in low-resource settings. Our primary objective was to develop and evaluate a measure of overall family functioning for the Kenyan setting, while also considering potential applicability to other locations with socioeconomic and cultural similarities. To do this, we employed a new combination of methods that allowed us to test a large pool of potential questionnaire items and establish criterion validity of the measure in a way that incorporated multiple family perspectives and a novel criterion reference—family clinical interviews by local interviewers. This methodology was designed to be flexible enough to use for validation of measures across other low-resource contexts and assessment topics as well.

## Methods

### Study Overview

**Figure 1:**
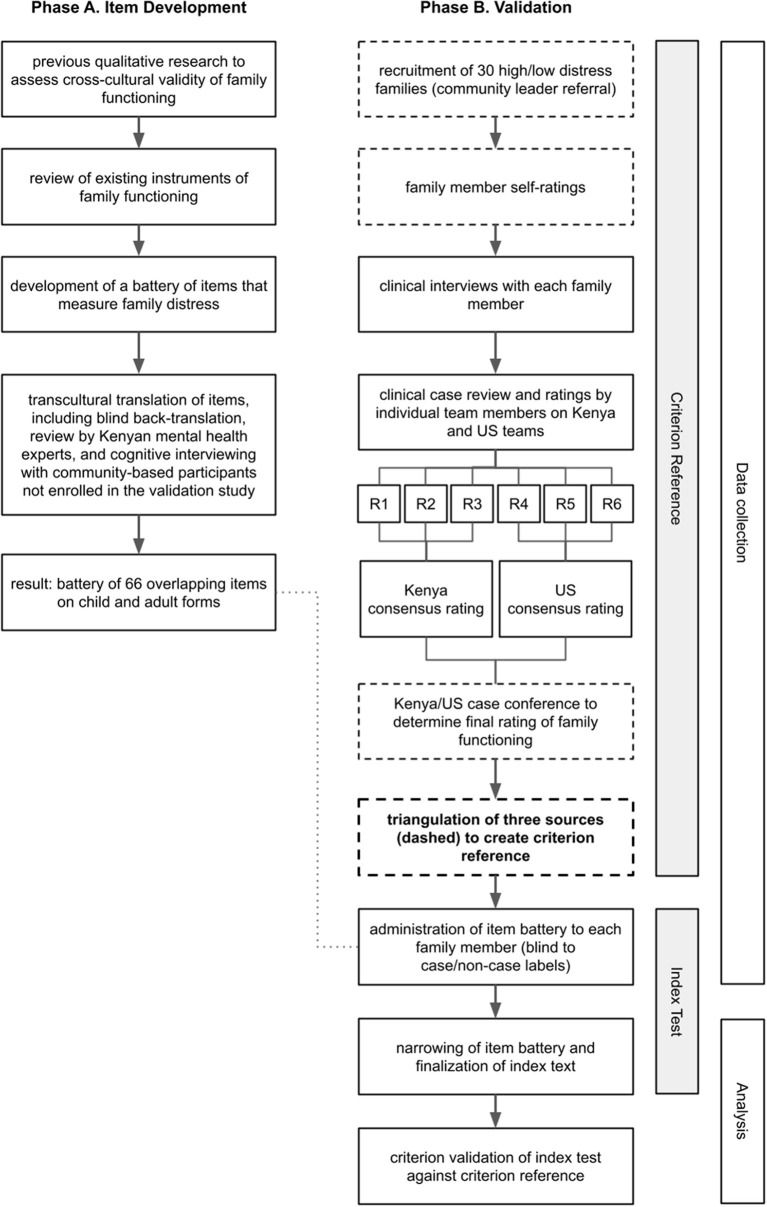
Study design and participant flow. In Phase A, we developed a comprehensive battery of items to assess family functioning. In Phase B, we constructed a final index test of items and evaluated its diagnostic accuracy for identifying family distress. Dashed boxes are sources for the criterion reference.

We conducted a two-phase, mixed-methods prospective diagnostic accuracy study to develop and evaluate a measure of family functioning. The study design and participant flow are shown in Figure 1. In Phase A, we developed a comprehensive battery of items to assess family functioning. In Phase B, we constructed a final index test of items and evaluated its diagnostic accuracy for identifying family distress. Our methodology draws upon several existing approaches that we modified and combined, as described below. This study protocol was reviewed and approved by the Duke University Institutional Review Board and the Institutional Research and Ethics Committee for Moi University in Kenya.

### Phase A Methods: Item Development

As theoretical constructs of family functioning and corresponding measures have been developed primarily in HICs, we first examined the extent to which the same constructs and existing measures were relevant and appropriate for use in Kenya and LMICs more broadly. We followed a process of cross-cultural validity appraisal similar to those used by Flaherty et al. (1988) and Kohrt et al. (2011). Our approach included: (1) collecting qualitative data to examine how family functioning is conceptualized in this Kenyan context; (2) comparing our findings to existing measures; (3) using results to develop a comprehensive battery of items; and (4) piloting all of the items using a transcultural translation process (Van Ommeren et al., 1999).

#### Step 1: Qualitative Data Collection and Analysis

We began by collecting and analyzing qualitative data to examine the concept of family functioning and its associations with mental health outcomes to identify risk and protective factors that could inform assessment and treatment. We first conducted nine interviews with individuals, both female (5) and male (4), who had experience in mental health care or in working with psychosocial programs for families. These were conducted by the lead author in English. We then conducted 15 focus group discussions (FGDs) that included groups of adolescents aged 12–17 years (Total *N* = 66; 42% female) and groups of adult family members (Total *N* = 54; 46% female) who cared for children in this age range. FGDs were led by Kenyan Research Assistants (RAs) in Kiswahi. FGD guides were semi-structured and focused on: (1) describing families who were functioning well vs. families experiencing distress and (2) describing child and adolescent outcomes associated with being in families in either category. Adults and adolescents were asked to discuss the questions and to engage in role plays to provide examples of family interactions. Interviews and FGDs were audio recorded and transcribed, with FGDs translated verbatim into English during transcription.

We conducted thematic content analysis through an in-depth grounded theory approach, using an inductive process (Strauss and Corbin, 1990). Analysis included: open coding to extract concepts and organize codes into categories for axial coding (Strauss and Corbin, 1990); condensing codes into the most salient categories; coding transcripts using an analysis program, Dedoose version 7.0.23, Dedoose (2016); and theoretical memoing (Birks et al., 2008). Results were synthesized by examining themes most salient to family functioning in Kenya, conserving Kiswahili terminology to reference for translations. Results informed the constructs we needed to measure. Focusing on these constructs, we used participants' descriptions to generate a comprehensive list of “local indicators” ranging from specific behaviors to broader descriptions of family characteristics important for family functioning. We created each indicator to be an appropriate length for a survey item (i.e., short sentence or phrase) so that the list became a guide for what might be important to represent in the items of a family functioning measure in this context.

#### Step 2: Comparison With Existing Measures

Next, we reviewed the content of several measures of family functioning developed primarily in HICs, and their theoretical underpinnings, to evaluate the fit with the local indicators generated from the qualitative data (measures listed in Supplementary Table 1). We evaluated construct validity by comparing themes identified from our qualitative work with the primary constructs assessed in the existing measures. We identified overlapping-constructs and mismatches-constructs on existing measures that did not emerge in our qualitative data—and “extra” constructs—constructs from our data that were “missing” from the measure.

At the item level, we then evaluated content equivalence by comparing our list of specific, data-driven indicators from qualitative findings to items on the existing measures to assess how well they matched in terms of their meaning and how salient they were to qualitative themes. To assess semantic equivalence, we focused on whether the wording of the items would be amenable to translation without completely changing them. This was influenced by factors such as whether items were worded or phrased in complex ways or dependent on idioms of distress not relevant across contexts. Based on these comparisons, we evaluated whether a given measure was an overall good match with the local understanding of family functioning across construct, content, and semantic characteristics. Throughout the process, we also gathered information related to each measure's previous use in research, existing data on psychometric properties, costs for use, and use or translations across cultures.

#### Step 3: Creating and Piloting the Battery of Items

After comparing local indicators with existing measures, we decided whether to adapt existing measure(s) and/or develop new items using the local indicators. This led to an initial battery of items. For each item, we completed a transcultural translation process (van Ommeren, 1999). This included blind back-translation, an external review by Kenyan mental health experts, and several rounds of one-on-one cognitive interviewing with community-based adolescent and adult participants to ensure comprehensibility, acceptability, relevance, and completeness. Each item was adapted iteratively until it fulfilled all of these criteria for at least three participants.

### Phase B Methods: Finalizing and Evaluating the Index Test

In Phase B, we trimmed the pool of 66 items into a shortened, final index test and evaluated its diagnostic accuracy. As part of this process, we recruited a community sample of families and constructed a criterion reference for family distress by triangulating data from family members, community leaders, and locally-conducted clinical interviews. We then identified which items in the full battery best discriminated between cases and non-cases and included the best items in the final index test. We evaluated the diagnostic accuracy of this index test against the criterion reference, generating cut-off scores indicating family distress.

#### Setting, Participants, and Recruitment

We recruited families from peri-urban communities outside of Eldoret, Kenya in 2016. Seven community leaders (e.g., village leaders and pastors) with knowledge of families in their jurisdiction were asked to recommend, in equal proportions, families who exhibited characteristics of high or low distress. To describe high family distress, leaders were asked to think of families who “need advising” related to family relationships. The leaders did not share names with the research team until after they asked the listed families if they were interested in learning more about the study. If a family expressed interest, the leader provided their contact information; a trained RA then approached the family to complete the informed consent and assent procedures (but remained blind to the leader's categorization of high/low distress). To be eligible, a family had to include at least one child between 8 and 17 years old and at least one caregiver above age 17. Caregivers were defined as any primary adult responsible for the child and did not need to be biologically related. In families with more than one eligible child, caregivers selected the child with the most suspected emotional or behavioral concerns. If caregivers did not have concerns about any child, they selected the one whose birthday fell next.

#### Construct a Criterion Reference

Lacking access to typical “gold standard” references often used in HICs, we had to establish a new criterion reference for this context before evaluating a measure. To do this, we collected and triangulated data from three sources: community leaders, self-ratings from family members, and clinical interviews.

##### Data Collection on Caseness

First, leaders who referred the families rated each family's overall functioning relative to other Kenyan families on a 4-point scale where “1” represented the worst functioning and “4” represented the best. When asked to rate, leaders were given broad examples of indicators drawn from the qualitative work (e.g., unity, how members talk together). Leaders gave their ratings to an RA in a sealed envelope to remain unknown to the research team until all other data were collected. Second, each family member used the same 4-point scale to provide a self-assessment of their family's functioning relative to others; this rating was distinct from the questionnaire but given at the same time before beginning the questionnaire. To give this rating, family members were asked to rate how well the family “relates to each other” compared with other families.

This process of seeking community nomination and self-assessment was a variation on the method of “known groups” validation (Murray et al., 2018). To strengthen this approach, we triangulated this data with a third source: clinical interviews by local interviewers. In this step, family members participated in individual clinical interviews with interviewers trained and supervised by psychologists from Kenya and the U.S. Interviewers were Kenyan and from the area; they included one masters-level psychologist and three lay interviewers without professional mental health training; two had Bachelor's degrees, and one had a 2-year diploma.

Interviews were recorded, transcribed, and read separately by study team members in Kenya and the U.S. Members independently rated each family using the Global Assessment of Relational Functioning (GARF; Group for the Advancement of Psychiatry Committee on the Family, 1996). The GARF, introduced in the fourth edition of the Diagnostic and Statistical Manual of Mental Disorders (4^th^ ed.; DSM-IV; American Psychiatric Association, 2000), was developed as an interpersonal relationship-based rating to mirror the Global Assessment of Functioning (GAF) used by mental health clinicians to rate the overall functioning of an individual on a scale from 1 to 99. For the GARF, clinicians assign a rating between 1 and 99 based on three domains that are common across multiple leading theories of family functioning: (1) Organizational Structure, (2) Emotional Climate, and (3) Problem-Solving/Interactional Skills. To guide ratings, we adapted the existing descriptions to incorporate specific indicators and examples from the qualitative data. For each family, raters in both locations recorded an overall numerical score and scores for each of the above GARF domains. Scores correspond to functioning descriptions using the following scale: Most Dysfunctional (1–20), Critically Dysfunctional (21–40), Somewhat Dysfunctional (41–60), Somewhat Functional (61–80), and Most Functional (81–99). Raters then discussed each case within country teams to determine a consensus rating. Once team consensus ratings were established, the Kenya and U.S. teams conducted a joint case conference via teleconference to determine a final consensus rating for each family. All raters were blinded to leader ratings and family self-report.

##### Classifying Cases/Non-Cases

We classified a family as a “case” of family distress by triangulating information from our three sets of informants: community leader referrals, family self-ratings from each member, and consensus ratings based on clinical interviews. The case criterion for leader referrals was a low functioning rating (1 or 2 on the 4-point scale). For family self-report, the case criterion was at least one member rating the family as low functioning (1 or 2). For clinical ratings, the criterion was a consensus score of 60 or less (mapping onto categories of Most, Critically, or Somewhat Dysfunctional). We classified families as follows: (1) a potential case if meeting the case criterion for 2 or 3 sources; (2) a non-case if meeting criterion for 0 sources; (3) or dropped from further analysis if meeting criterion for only 1 source.

#### Administering the Battery of Potential Items to Study Participants

Next, a team of Kenyan RAs (not involved with the interviews) administered the refined battery of items to each participant individually. Enumerators read aloud each item and recorded answers on a tablet computer (Again, the item battery was not used to determine caseness).

#### Analysis

We had two objectives in our analysis: (1) narrow the item battery and finalize the index test; and (2) assess the diagnostic validity of the final index test against the criterion reference.

##### Finalizing the Index Test

Adults and children answered 66 shared questions about their family's functioning. To select the final set of items, we combined two empirical approaches with our theoretical understanding of the construct. First, we used the technique of Goldberg (1972) to calculate gradient scores of item endorsement to identify items that best discriminated between cases and non-cases. For items with a 1–10 response scale, we defined endorsement as a response in the 6–10 range. For items with a 0–3 scale, we defined endorsement as a response in the 2–3 range. Items were reverse coded as needed so that higher numbers represented more family distress. Gradient scores were calculated for each item by taking the difference in the endorsement proportion by case status.

**Figure 2:**
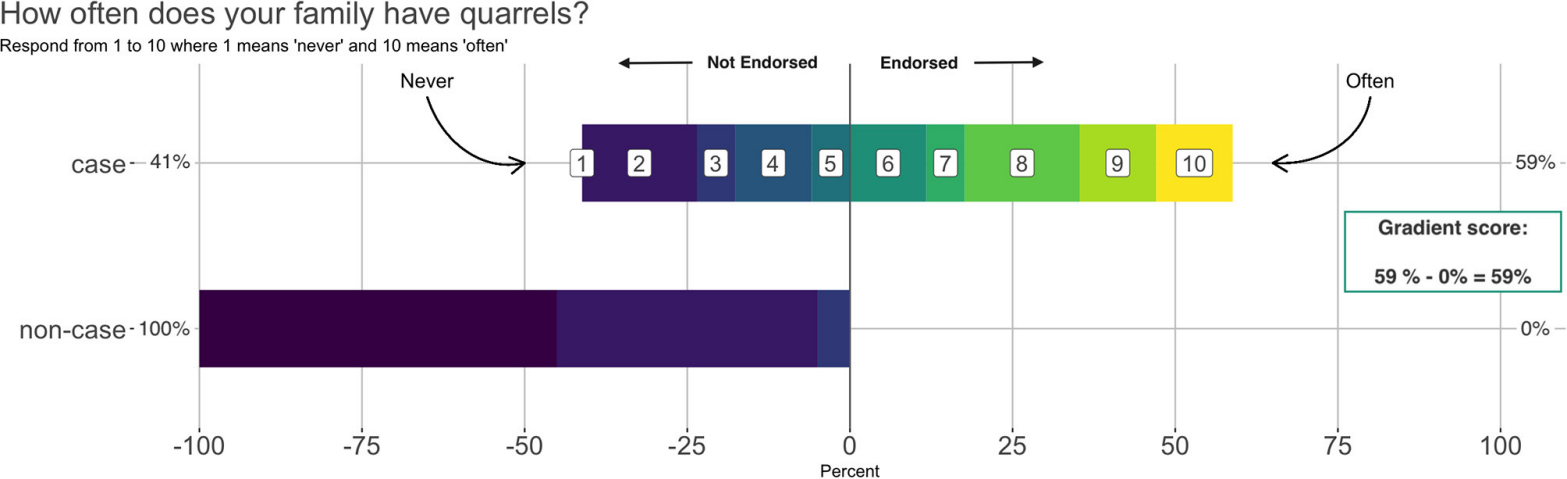
Example calculation of an item gradient score. This example shows that 59% of cases endorsed the item “How often does your family have quarrels?” by giving a response in the 6–10 range. None of the non-cases endorsed the item. Therefore, the gradient score for this item is 59% – 0% = 59%.

Figure 2 shows an example calculation. For instance, if 59% of cases responded to the question “How often does your family have quarrels?” with a response in the 6–10 range (indicating quarrels happen more often than not), this means that 59% of cases endorsed the item. If 0% of non-cases endorsed the same item with a response in the 6–10 range, this would be a gradient score of 59 – 0 = 59 percentage points (or a proportion of 0.59). Positive gradient scores indicate that items discriminate between cases and non-cases.

This example shows that 59% of cases endorsed the item “How often does your family have quarrels?” by giving a response in the 6–10 range. None of the non-cases endorsed the item. Therefore, the gradient score for this item is 59% – 0% = 59%.

For the second empirical method, we used the caret (Classification and Regression Training) package (version 6.0-85; Kuhn et al., 2017) in R (version 3.6.2; R Core Team, 2016) to identify which items are most important to predicting which families were labeled cases of family distress. We trained several classification models on the adult and child data (separately) with leave-one-out cross validation (5 repeats, 10 folds) and extracted the variable importance metric for each item across each model. From these results, we identified the top 20 items ranked as most important to the models' prediction of cases of family distress.

In machine learning literature, screening items like the ones we developed are referred to as “features,” and feature selection is the process of identifying which features (also known as predictors) to include in a classification model for optimal predictive performance (Kuhn and Johnson, 2019). The tree-based classifiers we used have built-in metrics of variable importance, so each item is automatically ranked in terms of its importance to the predictions. Items that help a model correctly “guess” whether or not a family was labeled a “case” or “non-case” receive a higher importance ranking. For instance, a model might learn that responses to the question, “How much love is your family?,” in combination with the other data, are associated with a family being labeled a case. Whereas knowing responses to: “How much do you agree that all members can be trusted with money?,” might not provide useful information to predict case labels. We examined each item's ranking across all models to identify the top 20 most important.

To finalize the index test, we combined the item gradient scores, information on item importance generated from the classification models, and our conceptual understanding of family functioning to select a subset of 30 items that made up the index text we sought to evaluate.

##### Assessing Diagnostic Validity of the Index Test Against the Criterion Reference

The final analysis was to estimate classification accuracy of the index test (adult and child versions) using the OptimalCutpoints package in R (version 1.1-4; López-Ratón et al., 2014). We opted to find the cut point for each version (adult and child) that equally optimized sensitivity and specificity for identifying families with potential distress.

## Results

### Phase A Results

#### Qualitative Findings (Step 1)

Themes that emerged from qualitative data reflected what was most salient for participants in differentiating families who are functioning well from families experiencing distress. The findings highlighted interaction patterns associated with negative family outcomes, including: conflict specific to roles and responsibilities; favoritism and discrimination often associated with overly harsh discipline; distance/mistrust across multiple relationships; and incomplete, avoidant, and/or aggressive communication processes during problem-solving, including lack of parental advising. Results also showed the importance of the corresponding positive interactions: agreement on roles and responsibilities, fairness and trust, collaborative problem-solving, clear communication, and spending positive time together. The concept of unity, or lack thereof, was often central to the conversations about overall family functioning; families with pervasive negative or distant patterns were described as “going their own way,” while those with overall positive relationships were referred to as “together.” Unsurprisingly, a common source of stress and conflict was the lack of financial resources, and issues related to gendered roles and power dynamics were prominent throughout the data.

#### Comparison of Local Qualitative Data With Existing Measures (Step 2)

##### Construct Validity: Did Constructs Match Qualitative Themes?

When comparing qualitative themes with existing measures, we found at least some conceptual overlap with all of the existing measures we reviewed. For instance, almost all included a construct related to family communication and problem-solving. Many also had a subset of matching constructs but with “extra” or “missing” constructs that led to an overall mismatch (e.g., missing the favoritism/discrimination theme from our findings). For most measures, creating an adequate match with our qualitative findings through the removal or addition of constructs would have been too extensive to maintain the integrity of the measure and its underlying theory.

The one assessment tool we examined that showed good construct validity was not a survey but a macro-level assessment tool based on clinician rating–the Global Assessment of Relational Functioning (GARF; Group for the Advancement of Psychiatry Committee on the Family, 1996) that is described above in the procedures for determining caseness. The three domains—organizational structure, emotional climate, and problem-solving—and their accompanying descriptions proved to be a very good fit with the themes and specific indicators of family functioning from qualitative data. While the GARF did not provide items to adapt, it applies a diagnostic approach to assessing relational problems that became the framework for the interview-based criterion reference (Notably, there have been self-report measures developed to accompany the GARF whose specific items showed the same challenges associated with other measures.).

##### Content Equivalence: Did Items on Existing Measures Match Local Indicators From Qualitative Data?

While constructs measured by some existing tools overlapped with qualitative themes, overall sets of items on existing measures showed a poor match with indicators generated from qualitative data. For example, while affective aspects of relationships and communication were important for existing measures and within local indicators, many items on existing measures ask respondents to reflect generally on communication about feelings, or about how they solve problems related to emotions. In contrast, local descriptions of affect and communication were more behavioral, more subtly reflecting emotional tone within communication (e.g., greeting each other happily, sitting and laughing together). This pattern of local indicators being more behavioral was present across multiple constructs.

##### Semantic Equivalence: Was Wording on Existing Measures Amenable to Cross-Cultural Translation?

Semantic equivalence varied greatly across items within most measures, with some simpler items very amenable to translation. However, almost all measures included multiple items that were worded or structured in ways that would make translation difficult, resulting in an item that hardly resembled the original. Some items included English-language idioms of distress, which of course facilitate understandability in the original language but pose a barrier to accurate translation. Many are also detailed and list many descriptors together in one item, resulting in a translation that becomes awkward and long; in an attempt to capture the full meaning, the item becomes nearly impossible to understand. If the items are shortened and simplified to be easily understood, the new items bear little resemblance to the originals.

#### Item Battery: Newly Developed Items (Step 3)

Given the shortcomings of existing measures in terms of transferability to the Kenyan context, we did not adapt any existing measures and instead generated a list of potential items from the local indicators derived from the qualitative data and theoretical considerations. The resulting battery included 66 items common to both the adult and child forms. Items broadly encompassed the areas of relational functioning that comprise the GARF domains: Organizational Structure, Emotional Climate, and Problem Solving.

#### Phase A Pilot Test Findings (Step 3)

As described above, all items were subjected to the iterative transcultural translation process, and all met criteria for inclusion in the final item battery. Response choices were also evaluated, and we determined that members of the target population were comfortable responding to questions by considering a 10-step ladder displayed visually. Most items were phrased as a question (e.g., “How often is there misunderstanding in your home?”) with response options on the 10-point scale (e.g., 1 = never, 10 = often), with some asking for level of agreement on this same scale. A few items focused on communication behaviors have a simple 3- or 4-point response scale. For instance, participants were asked “When your family has problems related to money, does your family…,” and response options included: “quarrel”, “not talk about it”, “talk calmly”, or “not a problem.” While we were hesitant to use different question types (e.g., frequency vs. agreement) and to incorporate different response options, we decided to maintain uniformity where possible but to ask questions in the way that made the most intuitive sense for that particular indicator, rather than forcing them into the same structure.

### Phase B Results

#### Participants

Thirty families: (a) were rated by the community leader who referred them, (b) completed the clinical interview, (c) provided self-reported ratings of family functioning, and (d) completed the full item battery. They included 44 adults with a mean age of 37.9 (*SD* = 8.7; 65.9% female) and 30 children with a mean age of 12.6 (*SD* = 2.6; 46.7% female). Almost half of families (47) had two adults, and average household size was 5.9 members (*SD* = 2.1). Mean weekly income was 2,107 Kenyan Shillings (approximately 21 USD; *SD* = 3,090 Kenyan Shillings). This is 30% below the 2015/16 national urban poverty line.

#### Cases and Non-Cases

**Figure 3:**
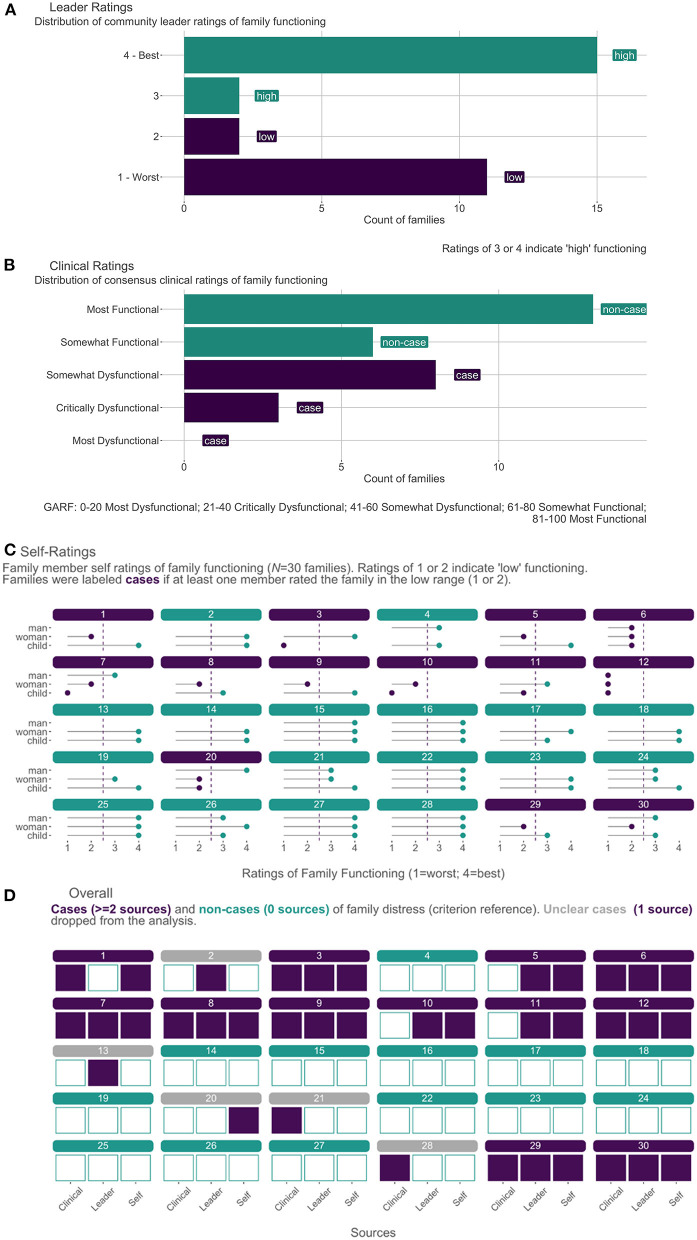
Establishing the Criterion Reference for Family Functioning (*N* = 30 families). **(A)** Distribution of leader ratings on a 4-point scale. **(B)** Distribution of consensus clinical ratings (0–100) grouped by GARF categories. **(C)**. Family member self-ratings on 4-point scale. **(D)** Final classification of cases and non-cases of family distress.

We identified possible cases of family distress according to each source and defined a “case” of family distress as meeting criteria according to two or more sources. Figure 3 shows ratings by source and summarizes how families came to be labeled as cases, non-cases, or unclear cases that were dropped from the analysis. As in Figure 3A, community leaders rated 13/30 families as distressed (i.e., potential case). This frequency was expected because we asked leaders to nominate well-functioning and distressed families in equal proportions (i.e., not indicative of community prevalence).

According to results of the clinical interviews shown in Figure 3B, the clinical team labeled 11/30 families as a potential case with consensus GARF ratings of “somewhat dysfunctional” (8/11) or “most dysfunctional” (3/11). Self-ratings from each participating family member are shown in Figure 3C. Overall, 13/30 families qualified as a potential case according to self-report because at least one family member rated the family as distressed. Among these 13 families, 3/13 had perfect agreement regarding distress among all participating members, and 8/13 met the threshold on the basis of one report of distress—6/8 of which came from female adults.

The three sources are triangulated in Figure 3D, showing that we ultimately labeled 12/30 families as cases because they met the threshold according to two sources (4/12 cases) or three sources (8/12 cases). Among the 4/12 families who met the case definition with 2 sources, the most common concordance was between leader and self-report. Of the remaining 18/30 families not labeled as a case, 13/18 had perfect concordance across sources as non-cases. The remaining five were classified as distressed by only one source and were dropped from further analysis. We conducted the remaining analysis with 26 of the original 30 families: 12 cases and 13 non-cases.

#### Finalizing the Index Test

**Figure 4:**
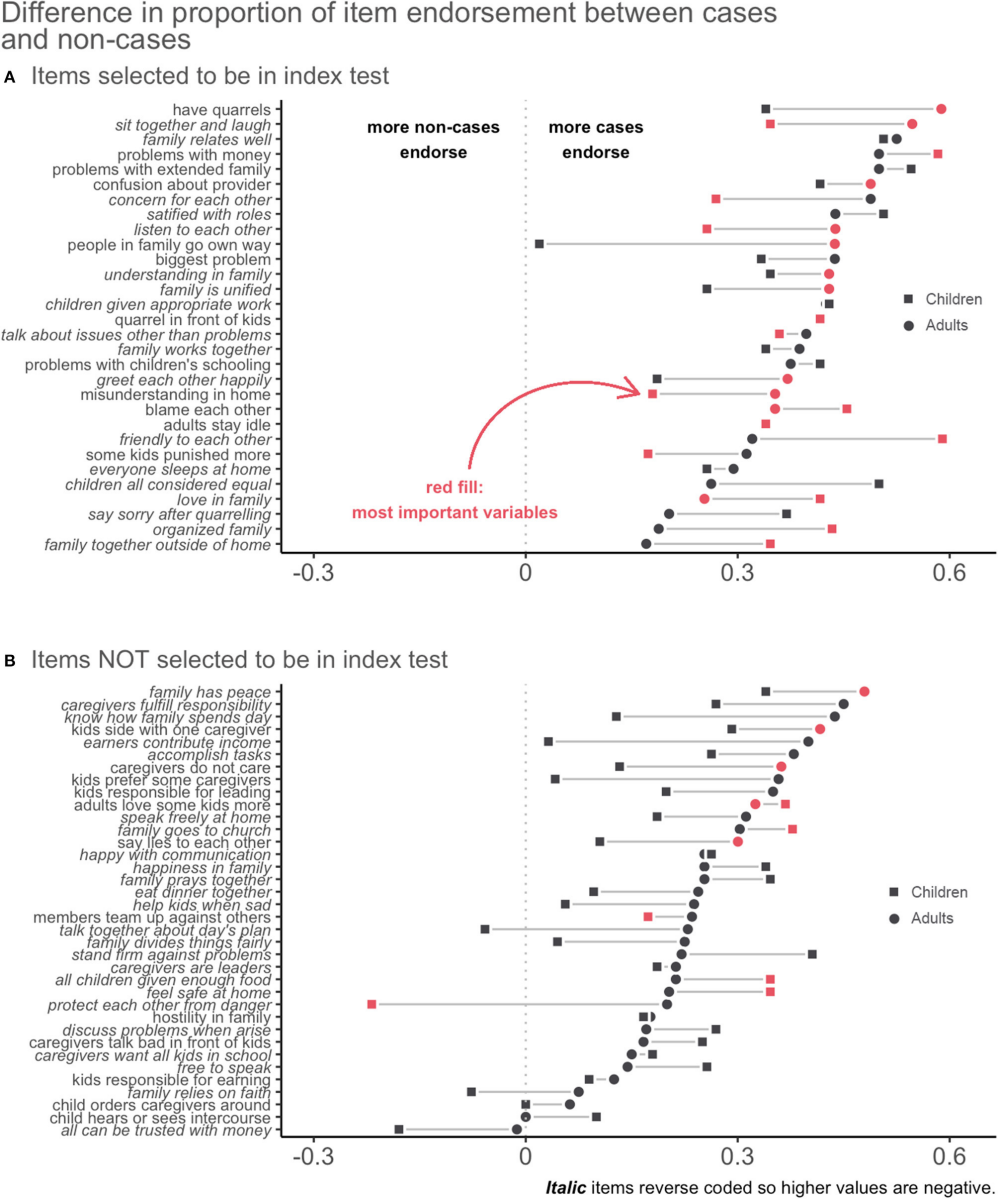
Gradient scores of family functioning items common to adults and children combined with indicators of variable importance. Lists the items selected **(A)** and not selected **(B)** to be in the final scale. The first item, “have quarrels,” had the highest gradient score for adults, meaning that answers to this item had the largest difference in endorsement by cases vs. non-cases. The gradient was smaller for children, but still large enough overall to make it an effective item.

As described above, we examined the extent to which each of the 66 items discriminated between family distress cases and non-cases as a first step in narrowing down the item battery. Results presented in Figure 4 show gradient scores for each item by reporter. Figure 4A shows the 30 items we selected for inclusion in the final index test based on empirical results and our theoretical understanding of family functioning in this context. As an example of the decision-making process, one item was “When things fail, how often do people in your family blame each other?” This item had large gradient scores according to both adult and child reports. For example, 35.3% of adults from families labeled as cases endorsed this item compared to 0% of adults from families labeled as non-cases, for a gradient score of 35.3%. This item similarly discriminated between cases and non-cases according to child-report with a gradient score of 45.5%. As indicated by the dark stroke for both informants, the item was also among the most important in the adult and child classification models. After reviewing these empirical results supporting the inclusion of this item, we considered it from a theoretical perspective and decided that blame was indeed among the most important constructs of family functioning. We followed this process of determining inclusion for each item in the final index test.

Figure 4B shows the items we did not include in the final index test. Some of these performed well but were of less theoretical interest. Others had similar rates of endorsement between cases and non-cases. A few even had negative gradient scores, meaning that more non-cases endorsed the item. Some also performed well for adults but not for children, or vice versa, and we eliminated those to choose items appropriate for both reporters. Table 1 presents the final 30-item measure—the *Family Togetherness Scale (FTS)*. We transformed all responses to have values ranging from 0 to 1, so the possible range of each scale was 0–30; higher scores represent more family distress.

**Table 1:** Family togetherness scale (FTS).

**Number**	**Item**
***How much…[Response Options: 1-10: “None” to “A Lot”]***	
1	How much love is in your family?
2	How much understanding is there in your family?
3	How organized is your family?
4	How friendly are the members of your family to each other?
5	How much concern do family members show for each other?
6	How satisfied are you with how roles and responsibilities are divided in your family?
***How much do you agree…[Response Options: 1-10: “Not at all”***	
***to “Completely”**]*	
7	Your family is together and unified.
8	Your family relates well.
9	People in your family go their own way.
10	In your family, you work together.
11	The children in your family are all considered equal.
12	Children in your family are given an appropriate amount of work for their age.
13	Some children in your family are punished more harshly than others after making same mistake.
***How often…[Response Options: 1-10 Scale: “Never” to “Very Often”]***	
14	When the people in your family greet each other, how often do they greet each other happily?
15	How often does your family do other tasks together outside the home compound?
16	How often does your family sit and talk about issues besides problems?
17	How often do all members of your family sleep at home at night?
18	How often does your family sit together and laugh during the evenings?
19	How often do people in your family listen to each other at home?
20	How often does your family have quarrels?
21	How often do you and your spouse quarrel in front of your children?
22	How often do people in your family say sorry to one another after quarreling?
23	When things fail, how often do people in your family blame each other?
24	How often is there misunderstanding in your home?
25	How often has there been confusion about who provides for your family?
26	How often do adults in your family stay idle?
***When your…[Response Options: “Not talk about it”, “Talk calmly”**,*	
***“Quarrel”, “You have not had the problem”**]*	
27	When your family has problems related to money…
28	When your family has problems related to children's school business…
29	When your family has problems related to extended family.
30	What is the biggest problem your family has had lately? When that happened…

*For the 10-point scale, a visual aid of a staircase was used during verbal administration*.

#### Diagnostic Validity

Estimates of diagnostic accuracy are shown in Table 2. The standardized alpha value for each scale was high, reflecting the fact that we chose to include some statistically redundant items for theoretical reasons. The optimal total score cutoff for family distress was 8.1 for adults and 8.9 for children. Overall, these cutoffs correctly identified distressed families in 89% of cases according to adult report and 76% of cases according to child report. The area under the curve estimates for adults and children suggest that both tests are good at distinguishing between cases and non-cases. The optimal cutoffs are associated with estimates of sensitivity/specificity of 0.88/0.90 for adult-report and 0.75/0.77 for child-report measures. Child and adult total scores are highly correlated (*r*_*child*−*adultman*_ = 0.86, *r*_*child*−*adultwoman*_ = 0.64).

**Table 2:** Diagnostic validity results.

	**Range**	**Cutoff**	**Alpha**	**False**	**False**	**Accuracy**	**Area under the curve**	**Sensitivity**	**Specificity**
				**positive**	**negative**		**(95% CI)**	**(95% CI)**	**(95% CI)**
Adult	0–30	8.1	0.96	2	2	0.89	0.918	0.88	0.90
							(0.825, 1.011)	(0.64–0.99)	(0.68–0.99)
Child	0–30	8.9	0.96	3	3	0.76	0.897	0.75	0.77
							(0.777, 1.018)	(0.43–0.95)	(0.46–0.95)

*CI, confidence interval*.

## Discussion

We developed and conducted an initial evaluation of a measure of family functioning in Kenya using a novel mixed-methods approach. Our aim was to apply empirical, quantitative methods alongside theoretical considerations and formative qualitative data to generate a screening measure with strong psychometric properties, including diagnostic validity. We developed items to be broad enough to apply to other settings—especially those with socioeconomic and cultural similarities—and we aimed for simplicity in item content and structure. This study responds to the dearth of validated family functioning assessment tools for LMICs.

Findings yielded a 30-item family functioning measure—the Family Togetherness Scale (FTS). This final group of items were: (1) among the most effective for differentiating families experiencing distress from those who were not and (2) representative of the most salient aspects of family functioning identified in qualitative work. Some items fall clearly within one of the three GARF domains: Organizational Structure (e.g., How satisfied are you with how roles and responsibilities are divided between members?); Emotional climate (e.g., How much concern do family members show for each other?); and Problem Solving/Interactional Skills (e.g., In your family, you work together.). Others conceptually relate to more than one, mirroring how they overlapped in the qualitative data. For example, items related to being “together and unified” and whether members “go their own way” capture overarching descriptions that could reflect difficulties in problem-solving, roles disorganization, and lack of emotional support. Similarly, “sit and laugh together” is an indicator of both emotional climate and interactional skills; this item is also an example of a very behavioral indicator derived directly from qualitative data in this context.

Overall, the *FTS* includes items that are broad and simple (e.g., “your family relates well”), as well as more specific items to explicitly address topics from our qualitative work. These topics included: (a) roles and responsibilities, (b) discrimination and favoritism related to treatment of children, and (c) specific context-salient stressors cited as most frequent causes of conflict and family distress: finances, children's school issues, and issues related to extended family. Both general and specific items may be relevant in other settings as well.

### Roles and Responsibilities

While delineation of roles is important across contexts, our qualitative findings, previous literature, and items that emerged in the validation process highlight the particular importance in low-resource settings. Because disagreements about roles often emerge when facing financial stress, the stakes for problem-solving around role assignment and fulfillment becomes high. Flexibility is needed as the family works to obtain resources (Black and Lobo, 2008), and gender norms impose even more complexity. For example, when men lack employment opportunities, they are less able to provide, and families may need to renegotiate traditional roles (e.g., women working outside of the home). This can strain the family system, and the FTS was designed to capture these struggles. For example, one item addresses “confusion about who provides,” and another asks generally about satisfaction with “how roles and responsibilities are divided.”

### Favoritism and Discrimination

Concern about discrimination and favoritism was prominent in qualitative findings and in items that emerged during quantitative selection. Previous literature presents reasons these inequalities may be particularly relevant to LMICs. First, poverty in general places extreme stress on families that forces difficult decisions about resource allocation (Bargain et al., 2014). Families also are more likely to include non-biological children due to disproportionate impacts of HIV, conflict, and other conditions elevating parental death and separation (Hapunda, 2015). Non-biological children are at heightened risk for unfair allocation of resources, traumatic experiences, and resulting mental health consequences (Morantz et al., 2013; Nduwimana et al., 2017). Third, gendered treatment is associated with discrimination, having potential negative consequences for girls or boys. Three items were developed to capture discrimination indicators, asking whether children are: considered equal, given appropriate work for their age, and punished with equal harshness.

### Specific Stressors Associated With LMIC Contexts

While we attempted to avoid items about circumstances affecting limited numbers of families, three stressors emerged very frequently in our Kenyan data that are also consistent with stressors documented in the broader LMIC literature: financial concerns, children's school issues (including fees), and challenges related to extended family. Participants described the possibility of severe conflict about these topics, as well as the benefits of open communication when facing these problems. As items about these topics then also emerged during the validity analysis, the measure includes very straightforward questions related to communication in response to these specific stressors, asking whether the family avoids talking about it, talks calmly, or “quarrels.” Whether these topics emerge as equally salient in other LMIC settings is a topic we hope to pursue in further research.

### Using the Family Togetherness Scale

The FTS has potential for both research and clinical use. First, it performed well in discriminating cases from non-cases in our Kenyan sample. We identified cut-off scores that identified families with probable functioning problems—as determined by our multi-method criterion reference—with sensitivity and specificity of 0.75 or greater. This suggests the FTS is a useful screener to identify cases and prompt further assessment. Using continuous FTS scores also may be a pragmatic option for tracking clinical progress and measuring intervention outcomes; larger-scale validation studies could evaluate these uses. Second, we expect that the FTS is broad and flexible enough to be applicable across many LMICs and perhaps some settings within HICs. We also expect it to be useful for different family structures, including families without children if parenting-related items are excluded. Both types of transferability need to be tested, however, taking into account that some cross-cultural translation is likely to be necessary in new contexts (Van Ommeren et al., 1999).

### Broader Applicability of Validation Methods

In this study, we demonstrate triangulating three sources of data—multi-informant family member self-ratings, community leader ratings, and consensus ratings from locally-conducted clinical interviews—to establish a criterion reference of family distress. We also demonstrate a method to combine empirical data on the prediction of caseness with theoretical grounding to identify items for an index test of family functioning. These methods may be beneficial in other low-resource contexts without typical “gold standards” and constraints that lead to smaller sample sizes (Also see Watson et al., 2020 for our related approach to adult mental health assessment).

## Strengths and Limitations

A major strength of this study is the use of community-based recruitment, detailed clinical interviews, and comprehensive self-report. This allowed us to triangulate sources of information and construct a credible criterion reference. The tradeoff for this level of detail was in time and resources for recruiting a large sample. The small sample size limited the extent of statistical analysis across types of validity. A larger sample would allow for: exploration of the structure of the FTS and factor analysis to generate subscales; subgroup analysis; more precision of sensitivity and specificity estimates; out-of-sample assessment; and improved generalizability. Nevertheless, this dataset yielded useful insights derived from the careful assessment. For instance, it is notable that the lower bounds on the confidence intervals of the diagnostic metrics are relatively high for the adult-report instrument given its intended purpose. We have less confidence in the child-report instrument, but this report gives others a good benchmark for further work.

Other limitations include not yet comparing the psychometric performance of this measure against other assessment tools and not being able to assess test-retest reliability. The sample also included only families that included children or adolescents aged 8 or above, and the qualitative work was limited to families that included adolescents. This precludes our ability to evaluate use of the FTS to assess functioning of families with younger children or without children; while we expect most items would be relevant, those that are parenting-related would need to be excluded for families including only adults. The general use of self-report is also an inherent limitation; recognizing this, we also developed an observational measure of family functioning (Giusto et al., 2019) that we began administering with the survey, though we do not have data on all participating families.

## Conclusions

Given the significant impact family functioning has on psychosocial outcomes for families and individuals, it is important to evaluate the family system with measures that are contextually relevant and validated. This is especially critical in LMICs where access to mental health resources and expertise in family treatment are scarce. The current study led to the development of a 30-item *FTS* useful for distinguishing families with and without relationship distress in a Kenyan sample.

## Data Availability Statement

The raw data supporting the conclusions of this article will be made available by the authors, without undue reservation.

## Ethics Statement

The studies involving human participants were reviewed and approved by Duke University Institutional Review Board/Moi University Institutional Review and Ethics Committee. Written informed consent to participate in this study was provided by the participants' legal guardian/next of kin.

## Author Contributions

EP conceptualized the study, led study implementation, and drafted the manuscript. AG contributed to study conceptualization and co-led implementation. AR contributed to drafting the manuscript. EF contributed to conceptualization and implementation and reviewed the manuscript. DA co-led conceptualization, contributed to implementation, and reviewed the manuscript. EG contributed to conceptualization, led statistical analysis, and contributed to drafting the manuscript. All authors contributed to the article and approved the submitted version.

## Conflict of Interest

The authors declare that the research was conducted in the absence of any commercial or financial relationships that could be construed as a potential conflict of interest.
