# Supplementary material for: Development of the Family Togetherness Scale: A Mixed-Methods Validation Study in Kenya
Source: Front Psychol. 2021 Jun 8;12:662991. doi: 10.3389/fpsyg.2021.662991 (PMC8217654; doi:10.3389/fpsyg.2021.662991)
Supplement: Supplementary file 1 [file Table_1.DOCX]

| **Supplementary Table 1**  ***Assessment Measures Reviewed*** | |
| --- | --- |
| **Measure** | **Citation** |
| Family Adaptability and Cohesion Evaluation Scale  IV (FACES IV) | Olson DH, Gorall DM, Tiesel JW. FACES-IV package: Administration. Minneapolis, MN: Life Innovations, Inc; 2006. |
| Family Adaptability, Growth, Partnership, Affection, Resolve (APGAR) | Smilkstein, G. (1978). The family APGAR: a proposal for a family function test and its use by physicians. J family practice, 6(6), 1231-9. |
| Family Assessment Measure-III (FAM-III) | Skinner, H.A., Steinhauer, P.D. and Santa-Barbara, J. (1995). Family Assessment Measure – III Manual. Toronto, Canada: Multi Health Systems. |
| Family Events Scale | Oregon Social Learning Center (1985). Family Events Scale (FMEVE). Unpublished instrument, Oregon Social Learning Center, Eugene, OR. |
| Family Relationship Index | Holahan, C. J., & Moos, R. H. (1983). The quality of social support: Measures of family and work relationships. British Journal of Clinical Psychology, 22, 157-162. |
| Global Assessment of Relational Functioning (GARF) | Group for the Advancement of Psychiatry Committee on the Family. (1996). Global assessment of relational functioning scale (GARF): I. Background and rationale. *Family Process*, *35*(2), 155-172. |
| How is Your Family Scale | Kellog Foundation. 1996. Familia y adolescencia: Indicadores de salud. Manual de Aplicación de Instrumentos. Washington, D.C. http://www.paho.org/Spanish/HPP/HPF/ADOL/Family.pdf |
| Index of Family Relations | Hudson, W. W. (1997). *The WALMYR Assessment Scales Scoring Manual*, Tallahassee, FL: WALMYR Publishing Company. |
| McMaster Family Assessment Device (FAD) | Epstein, N. B., Baldwin, L. M., Bishop, D. S. (1983). The McMaster family assessment device. Journal of Marital and Family Therapy. 9, (2), 171-180. |
| Scale for Assessment For Family Enjoyment Within Routines (SAFER) | Scott, S. & McWilliam, R.A. (2000). Scale for Assessment of Family Enjoyment within Routines (SAFER). Frank Porter Graham Child Development Center University of North Carolina At Chapel Hill: Chapel Hill, NC. |
| Scale for Sub-dimensions of Family Relations | Melgarejo[AG1] , M. C. (2008). Construcción y validación de una escala que mide funcionamiento familiar. Tesis de Maestría. Crisol, Centro de Posgrado en Terapia Familiar. Cuernavaca, Morelos, México. |
| Self-Report Family Inventory | Beavers, W. R., & Hampson, R. B. (1990). *Successful families: Assessment and intervention*. WW Norton & Co. |
